# Awards for Valuable Services in Connection with the War

**Published:** 1917-03-03

**Authors:** 


### ROYAL RED CROSS.

### Awards for Valuable Services in Connection with the War.

The Supplement to the *London Gazette* of February 23 contained the following :—

War Office, February 23.

The King has been graciously pleased to award the Royal Red Cross Decoration to the following ladies, in recognition of their valuable services in connection with the war :—

FIRST CLASS.

C. Alcock, Principal Matron, T.F.N.S., 5th Southern Gen. Hosp., Southsea.

M. L. T. Babb, Sister, Q.A.I.M.N.S.R., Mil. Hosp., Dover; M. Banfield, Matron, Q.A.I.M.N.S.R., Lord Derby War Hosp., Warrington; M. Bayldon, Matron, T.F.N.S., 4th Northern Gen. Hosp., Lincoln; H. Bigg, Matron, Charing Cross Hosp.; I. C. Bennett, Matron, Met. Hosp., Kingsland Road, London; A. M. Bird, Matron, Gt. Northern Central Hosp.; L. Bradburne, Matron, Meath Aux. Hosp., Dublin; S. A. Brown, Matron, Q.A.I.M.N.S.R., Dartford War Hosp.; A. C. G. Buller, Administrator, Grouped Aux. Hospitals, Exeter.

M. Carruthers, Matron, Q.A.I.M.N.S.R., Pavilion and York Place Hospitals, Brighton; L. O. Carter, Matron, T.F.N.S., 2nd Eastern Gen. Hosp., Brighton; H. Casault, Matron, Canadian Nursing Serv.; H. M. Cave, Matron, Bury and West Suffolk Hosp., Bury St. Edmunds; R. E. Crowdy, A.R.R.C., Principal Comdt., V.A.D.s, France; L. E. Cushon, Principal Matron, British Red Cross Hosp., Netley.

I. Davidson, Matron, Edinburgh War Hosp., Bangour; G. T. Davis, Matron, 1st Western Gen. Hosp., Fazakerley, Liverpool; E. Dodds, Matron, Bethnal Green Mil. Hosp., Cambridge Heath, E.; A. Dowbiggin, Matron, Mil. Hosp., Edmonton; E. Eddison, Matron, Royal City of Dublin Aux. Hosp.

D. Finch, Matron, University College Hosp., London; E. E. Fletcher, Matron, 2nd Western Gen. Hosp., Manchester; G. Fletcher, Matron, Q.A.I.M.N.S., Richmond Mil. Hosp., Grove Road, Richmond, Surrey; M. A. Foggett, Matron, Bradford War Hosp., Yorks.

G. R. Hale, Matron, Endell Street Mil. Hosp., London; M S. Hamer, Matron, Norfolk War Hosp., Thorpe, Norwich; H. Hannath, Matron, T.F.N.S., 5th Northern Gen. Hosp., Leicester; H. Hare, Matron, Q.A.I.M.N.S., Mil. Hosp., Grantham; M. L. Harris, Sister (Acting Matron), Q.A.I.M.N.S., Mil. Hosp., Felixstowe; L. V. Haughton, Q.A.I.M.N.S., Nursing Board and Matron, late Guy's Hosp., London; Hezlett, Matron, Richmond Aux. Hosp., Dublin; P. Hill, Matron, Adelaide Aux. Hosp., Dublin.

E. W. Jayne, Sister, Q.A.I.M.N.S.R., Barnet War Hosp., Herts; R. L. Jones, Officers' Hosp., Dublin.

A. M. Kellett, Matron, Aust. Army Nursing Serv.; A. E. Kerslake, Matron, T.F.N.S., 1st Southern Gen. Hosp., Edgbaston, Birmingham; F. Knowles, Matron, T.F.N.S., 4th London Gen. Hosp., King's College, London.

A. Lloyd-Still, Principal Matron, T.F.N.S., 5th London Gen. Hosp., St. Thomas's Hosp., London.

M. O'C. McCreery, Sister (Acting Matron), Q.A.I.M.N.S., Mil. Hosp., Cork; A. Macdonald, Acting Matron, T.F.N.S., 1st Eastern Gen. Hosp., Cambridge; E. Macfarlane, Sister (Acting Matron), T.F.N.S., Malta; M. McGivney, Matron, Mater Misericordiæ Hosp., Dublin; A. McIntosh, Matron, St. Bartholomew's Hosp., London; J. P. MacLeod, Matron, N.S.R., North'd War Hosp., Gosforth, Newcastle-on-Tyne; F. Macpherson, A.R.R.C., Q.A.I.M.N.S., Acting Matron, Mil. Hosp., Sutton Veny; M. McDougall, Staff Nurse (Acting Matron), T.F.N.S., Malta; C. Metcalfe, Matron, Graylingwell War Hosp., Chichester; C. W. Millar, Matron, T.F.N.S., 2nd Scottish Gen. Hosp., Craigleith, Edinburgh;

(*Continued on page vi.*)

